# Pressure Overload and Right Ventricular Failure: From Pathophysiology to Treatment

**DOI:** 10.3390/jcm12144722

**Published:** 2023-07-17

**Authors:** Nicolas Dayer, Zied Ltaief, Lucas Liaudet, Benoit Lechartier, John-David Aubert, Patrick Yerly

**Affiliations:** 1 Department of Cardiology, Lausanne University Hospital and Lausanne University, 1011 Lausanne, Switzerland; patrick.yerly@chuv.ch; 2 Department of Adult Intensive Care Medicine, Lausanne University Hospital and Lausanne University, 1011 Lausanne, Switzerland; zied.ltaief@chuv.ch (Z.L.); lucas.liaudet@chuv.ch (L.L.); 3 Department of Respiratory Medicine, Lausanne University Hospital and Lausanne University, 1011 Lausanne, Switzerland; benoit.lechartier@chuv.ch (B.L.); john-david.aubert@chuv.ch (J.-D.A.)

**Keywords:** right ventricular failure, right heart failure, adaptive hypertrophy, maladaptive hypertrophy, right ventricular–pulmonary artery coupling, pressure–volume loops, hemodynamics, echocardiography, therapy

## Abstract

Right ventricular failure (RVF) is often caused by increased afterload and disrupted coupling between the right ventricle (RV) and the pulmonary arteries (PAs). After a phase of adaptive hypertrophy, pressure-overloaded RVs evolve towards maladaptive hypertrophy and finally ventricular dilatation, with reduced stroke volume and systemic congestion. In this article, we review the concept of RV-PA coupling, which depicts the interaction between RV contractility and afterload, as well as the invasive and non-invasive techniques for its assessment. The current principles of RVF management based on pathophysiology and underlying etiology are subsequently discussed. Treatment strategies remain a challenge and range from fluid management and afterload reduction in moderate RVF to vasopressor therapy, inotropic support and, occasionally, mechanical circulatory support in severe RVF.

## 1. Introduction

The function of the right ventricle (RV) remained enigmatic for medicine and science for centuries. In 1628, Sir William Harvey wondered why nature provided human bodies with a ventricle dedicated to the sole purpose of nourishing the lungs, whereas all other organs receive blood from the left ventricle (LV). The conundrum persisted during the first half of the 20th century after experiments in dogs showed almost unchanged blood pressure and cardiac output after complete RV-free wall cauterization [1]. In addition, RV bypass with direct connection of the superior and inferior venae cavae to the pulmonary arteries, designated as the Fontan procedure, improves survival in patients with tricuspid atresia or single-ventricle anatomy [2].

Although hidden at rest, RV functionality reveals itself during exercise. Despite offering long-term palliation for specific congenital heart diseases, patients with Fontan physiology also have 35–60% less maximal aerobic capacity than healthy subjects and a markedly reduced maximal systemic venous return [3,4]. Indeed, the necessary increase in cardiac output required during exercise cannot take place without increasing transpulmonary driving pressure and, hence, central venous pressure with Fontan circulation. In turn, the pressure gradient between mean systemic filling pressure and central venous pressure decreases along with venous return. From this observation, it can be inferred that the RV’s main task is to increase both pulmonary artery pressure (PAP) and flow without letting the right atrial pressure (RAP) rise in situations of increased venous return [4,5,6,7]. 

Under physiologic conditions, the resistance opposed by pulmonary vessels to flow is very low, and mean pulmonary artery pressure (mPAP) rises <3 mmHg with cardiac output increasing by 1 L/min in subjects < 60 years old [8]. Consequently, the RV does not need as much capacity as the LV to generate pressure, which is reflected in many aspects of its anatomy and function. With its triangular geometry in the long axis and crescentic shape in the short axis, the RV cannot efficiently concentrate its force vectors towards the middle of its cavity like the LV. Furthermore, its walls are thin and have less muscularity, with only two layers of muscles instead of three in the LV. It also contracts sequentially in a kind of peristaltic movement, with the infundibulum already relaxing when the outflow tract is still contracting [7,9,10]. 

With this background, it is not surprising that RV failure mainly occurs in the setting of increased RV afterload and, hence, in the context of pulmonary hypertension (PH) [11,12]. Moreover, in pulmonary arterial hypertension (PAH) and chronic thrombo-embolic pulmonary hypertension (CTEPH), patients’ outcomes are predominantly determined by the response of the RV to increased afterload, hence by the RV systolic function [13,14]. Nevertheless, despite the RV’s utmost prognostic role in all PH conditions, there is so far no validated intervention targeted at preserving its function, and all therapeutic strategies proposed in PH essentially focus on afterload reduction.

The aim of this article is to review the pathophysiological mechanisms leading to RV failure, to understand how RV adaptation/maladaptation to its afterload can be assessed at bedside and to present the principles of RV failure therapy.

## 2. Determinants of RV Systolic Function and Coupling

During ejection, the RV reduces its volume by contracting its free wall towards the interventricular septum and by shortening its long axis [15], allowing blood transfer towards the pulmonary arterial tree with minimal backward regurgitation if the tricuspid valve is held closed. The ensuing stroke volume depends on the intrinsic ability of the RV muscle to generate force (contractility) and on preload and afterload as extrinsic factors. Of note, all these factors can be assessed on pressure–volume loops [7]. 

**Figure 1:**
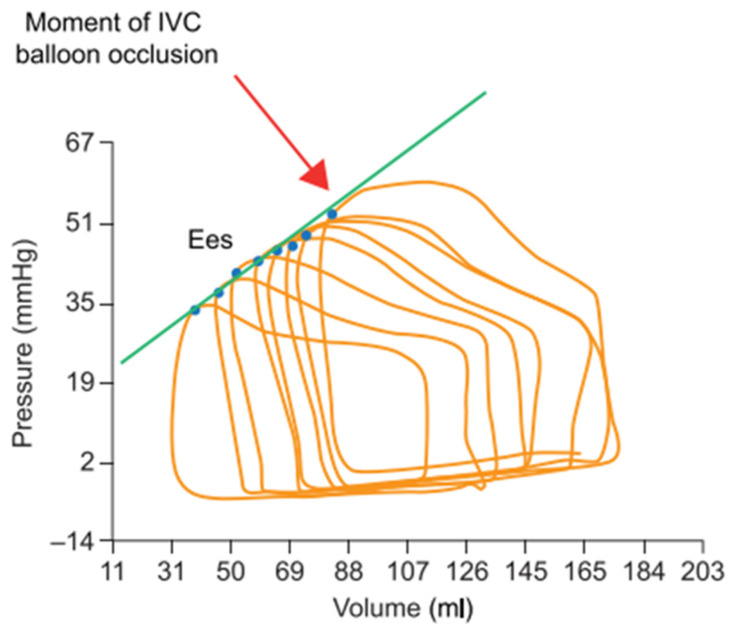
RV pressure–volume loops generated during progressive preload reduction (progressive occlusion of the inferior vena cava (IVC) with a balloon). The blue points indicate the maximal pressure/volume ratio (=end-systolic elastance, or E_es_) of each loop, and the green line shows the linear regression of the relationship between volume and pressure at all E_es_ (end-systolic pressure–volume relationship) to assess contractility. Adapted with permission from Tello et al., BJP 2019, John Wiley and Sons (Hoboken, NJ, USA). [16] Copyright © 2019, John Wiley and Sons.

Preload defines the amount of stroke volume recruited by the myocardial stretch before contraction according to the Frank–Starling law of the heart. It essentially accounts for the end-diastolic ventricular volume as a surrogate of fiber elongation and is mainly determined by the systemic venous return. Provided that contractility and afterload remain constant, an acute reduction in preload is matched by a predictable stroke volume decrease (Figure 1) [16].

During systole, highly contractile ventricles generate more pressure and reduce their volume more extensively than poorly contractile ventricles. Consequently, contractility correlates well with the maximal pressure/volume ratio, or maximal elastance (E), that occurs at the end of ejection (E_es_). On a single beat, E_es_, however, also depends on the actual preload and afterload conditions and cannot be considered an independent surrogate of contractility. Nevertheless, as E_es_ is the only elastance value to be shared by multiple ejecting beats at variable preloads and fixed afterloads, linear regression connecting these pressure–volume points (designated as the end-systolic pressure–volume relationship, or ESPVR) can be performed to assess contractility as a relatively load-independent measurement. ESPVR can thus be measured over multiple beats (PV loops family) recorded during slow balloon inflation in the inferior vena cava or during a progressive Valsalva maneuver (Figure 1) [17,18,19,20,21]. 

**Figure 2:**
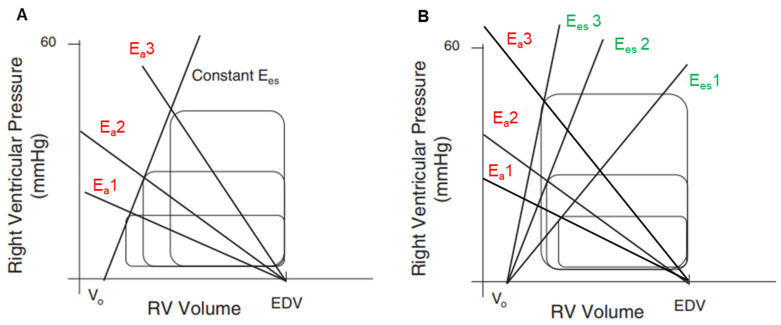
(**A**). RV pressure–volume loops model demonstrating stroke volume reduction with the increase in arterial elastance (E_a_) (E_a_1 > E_a_2 > E_a_3) at constant preload (unchanged end-diastolic volume) and constant contractility (linear relationship between all E_es_); RV-PA coupling decreases from E_a_1 to E_a_3. (**B**). RV pressure–volume loops model with increasing contractility (E_es_1 > E_es_2 > E_es_3) in response to increasing arterial elastance (E_a_1 > E_a_2 > E_a_3). In this model, stroke volume increases, indicating better coupling with E_es_3/Ea3 than with E_es_1/E_a_1. E_a_ = arterial elastance; EDV = end-diastolic volume; E_es_ = end-systolic elastance.

In the right-sided circulation, the resistive and pulsatile components of the hydraulic load opposed by the pulmonary circulation to RV ejection can be represented by a single factor: effective arterial elastance (E_a_). E_a_ accounts for net vascular stiffness, is independent of preload and contractility, and can be approximated by the ratio between the end-systolic pressure and stroke volume on pressure–volume loops (Figure 2). This simple afterload measurement has been validated through comparisons with input impedance spectra and arterial compliance [22,23]. By sharing the same units with E_es_ (mmHg/mL), E_a_ offers the possibility to assess how RV contractility may be adapted or not to afterload. Indeed, the ratio of elastances (E_es_/E_a_), referred to as RV-PA coupling, can be seen as the matching of the heart with its arterial circuit. Optimal E_es_/E_a_ values related to optimal energy transfer from the RV to the arterial tree lie between 1.5 and 2.0. In PH, RV volume increases when E_es_/E_a_ is <0.8, and RV-PA coupling is a main determinant of outcome [13].

## 3. RV under Pressure: Homeometric Adaptation

According to the PV loop family model, the RV is extremely sensitive to hydraulic load, and a non-counteracted increase in RV afterload (=E_a_ increase) results in a proportionate decrease in stroke volume [7] (Figure 2A). Indeed, experiments in dogs have shown that an acute increase in mPAP from 10 to 30 mmHg caused by pulmonary artery constriction reduces stroke volume by ~30%, whereas a rise in mean aortic pressure from 100 to 140 mmHg caused by aorta constriction only reduces LV stroke volume by ~10% [24]. Such a severe RV-PA uncoupling with highly depressed stroke volume does, however, not occur in most clinical conditions associated with chronic PH, at least at the onset of the disease. The increase in hydraulic load is usually progressive enough to let the RV adapt by increasing E_es_, which may preserve stroke volume and keep the E_es_/E_a_ ratio between 1.5 and 2.0 for some time (Figure 2B). E_es_ rise can, however, usually not exceed four to five times its basal value [25]. This first-line RV adaptation to afterload is called homeometric adaptation or Anrep mechanism. Of importance, it may fail in situations where pulmonary pressure rises very rapidly, like in massive pulmonary embolism, which may rapidly lead to low cardiac output, cardiogenic shock and, eventually, death [26].

### 3.1. Adaptive Mechanisms to Increased Afterload

In the context of progressively rising vascular load, RV E_es_ mostly increases through concentric muscle hypertrophy. By adding supplementary sarcomeres to existing ones in parallel, concentric hypertrophy enhances RV contractility and wall thickness while minimizing RV cavity dilatation at the same time. By raising the mass-to-volume ratio, the benefit of this response is to limit the increase in wall stress and associated O_2_ demand [25,27]. Of note, adaptive concentric hypertrophy occurring in pressure-overloaded RVs does never completely and successfully control wall stress, as opposed to the LV, which can usually normalize wall stress by sufficient concentric mass growth when it faces pressure overload, like in aortic valve stenosis [28].

Adaptive RV hypertrophy implies deep changes in all components of the RV myocardium, namely cells, extracellular matrix (ECM) and microvasculature. In cardiomyocytes, protein synthesis pathways are activated by two distinct processes: (1) local stress sensing by integrins, stretch-activated ion channels and titin [29] and (2) the up-regulation of the sympathetic nervous system (SNS) and the renin–angiotensin–aldosterone system (RAAS) [27,30,31,32]. Integrins are membrane-crossing heterodimers, attached to the ECM at one extremity and to the cardiomyocyte cytoskeleton at the other. They change conformation with increased wall tension and transduce mechanical stress to the nucleus through various signaling pathways (focal adhesion kinases and small GTPases) [33]. Titin is a giant protein that stabilizes myosin heavy chains in the sarcomeres and connects them to the Z-disc, conferring thereby most of the passive elasticity properties to cardiomyocytes. According to mechanical stress, titin may also uncover binding regions for signaling molecules that modulate its elasticity and interact with hypertrophy signaling [34].

In PAH, RAAS activation occurs both locally through increased wall stress and systemically through renal hypoperfusion [30], whereas SNS activity is enhanced through mechano-receptors located mainly in the aorta and the carotid arteries [35]. RAAS- and SNS-induced hypertrophy have been mostly studied in the LV. With the RAAS, signaling occurs through the activation of the angiotensin 2 type 1 receptor, a Gq protein-coupled receptor with downstream stimulation of multiple pathways including Ca^++^ mobilization, activation of protein kinase C, MAP kinases, tyrosine kinases and nicotinamide adenine dinucleotide phosphate (NADPH) oxidases (NOX), all of which regulate transcription factors associated with cardiac hypertrophy [36]. On the other hand, chronic catecholamines binding to β_1_-adrenergic receptors induce cardiac hypertrophy through the activation of nuclear protein kinase A and subsequent phosphorylation of nuclear targets that further enhance gene transcription [37]. In addition, cytoplasmic PKA activation by β-adrenergic receptor stimulation up-regulates inotropy through the phosphorylation of key molecules that increase either Ca^++^ availability or Ca^++^ responsiveness of the contractile system [38]. This mechanism plays a major role in enhancing RV contractility in cases of acute PAP increases. 

In adaptive RV hypertrophy, little expansion of the ECM also occurs to enhance mechanical resistance to increased wall stress and to scaffold new vessels growing around hypertrophied cardiomyocytes, but fibrosis is minimal [39]. New vessel growth is also necessary to provide enough O_2_ supply to larger cardiomyocytes and occurs through the effects of the vascular endothelium growth factor (VEGF) and angiopoietin-1 secreted by cardiomyocytes [40].

### 3.2. Transition from Adaptive to Maladaptive Hypertrophy

Although adaptive hypertrophy may preserve cardiac output and limit exercise intolerance in the early phase of most PH conditions, a chronic increase in RV afterload almost inevitably leads to RV failure [41]. This evolutive pattern may either reflect the progressive nature of pulmonary vascular lung disease, which may indefinitely increase RV afterload and finally overcome RV adaptation capacity, or, in most situations, reflect the inability of the RV to sustain long-term pressure overload and hence transition from adaptive to maladaptive hypertrophy. Although the exact mechanisms underlying this transition are not completely understood, both disease-specific aspects and mechanisms common to all PH conditions play a role [27,31]. 

Among the disease-specific factors, it is worth mentioning that *BMPR2* mutation carriers and scleroderma patients with PAH display reduced afterload-adjusted RV ejection fraction [42] and depressed sarcomeric function [43], respectively, compared to patients with idiopathic PAH, whereas patients with Eisenmenger syndrome have higher RV contractility and less diastolic stiffness in comparison to those with idiopathic PAH [44]. These mechanisms may account for much of the very different survival observed between these three etiologies in PAH registries (85% 3-year survival with PAH associated with Eisenmenger syndrome, 63% 3-year survival with idiopathic PAH and 52% 3-year survival with scleroderma-associated PAH in the ASPIRE registry) [45].

Factors generally associated with the transition from adaptive to maladaptive hypertrophy include deep metabolic perturbations and fibrosis, but also ischemia and excessive neurohormonal chronic overactivation, whose involvement is important regarding RV failure therapeutic options. 

Whereas normal RVs derive the majority of their energy production from lipid oxidation, pressure-overloaded RVs evolving towards failure increasingly rely on glucose metabolism [46], as evidenced by their elevated ^18^F-FDG uptake on positron emission tomography imaging [47]. However, instead of entering the oxidative pathway in mitochondria, glucose metabolites rather follow the glycolytic pathway, with final transformation into lactate and reduced ATP production [48]. As the glycolytic metabolism is insufficient to fulfill hypertrophic RV O_2_ demand, RV function may decline as a result of energy starvation. Interestingly, the therapeutic restoration of a better coupling between glycolysis and the mitochondrial oxidative pathway improved RV contractility in several animal models of PH [49].

The increasingly apparent fibrosis in failing RVs may be another important factor for systolic dysfunction [50]. With the deposit of ECM between muscular bundles (interstitial fibrosis), fibrosis creates zones of slow electrical conduction and prolonged action potential that may account for regional contraction inhomogeneity, intraventricular dyssynchrony and, ultimately, depressed RV contractility [51]. In addition, the high collagen content of interstitial fibrosis lessens RV myocardium elasticity and transforms an initially highly compliant organ into a stiff one [52], eventually contributing to elevated venous pressure and systemic congestion. At the cellular level, excess ECM production is the result of the excessive differentiation of myocardial fibroblasts into myofibroblasts due to prolonged wall stress and prolonged neurohormonal myofibroblast stimulation [39].

In addition, ischemia seems to play a major role in the reduction in RV contractility with time. As discussed above, enhanced RV wall stress is paralleled by increased myocardial O_2_ demand, but concurrent O_2_ supply may not fulfill O_2_ demand. First, all PH conditions lessen flow in the right coronary artery during systole by decreasing the pressure gradient between the aorta and the RV, and this may further worsen in cases of systemic hypotension [53]. Moreover, a mismatch between cardiomyocytes and capillary growth occurs with time in hypertrophic RV due to the downregulation of VEGF expression [54]. Indeed, VEGF is usually enhanced by hypoxia-inducible factor-1 (HIF-1), but it may also be turned off if oxidative stress in mitochondria is high enough to deactivate HIF-1, which is the case in maladaptive hypertrophy [48]. In addition, hypoxemia induced by the disease leading to PH, decreased O_2_ venous saturation on low cardiac output and the re-opening of the foramen ovale may also concur with ischemia. Besides reducing RV contractility, ischemia may also be a major contributing factor to fibrosis and apoptosis in failing RVs.

Finally, chronic SNS overstimulation leads to the downregulation of sarcolemmal β-adrenergic receptors, with loss of stimulation of the hypertrophic pathways, loss of inotropic stimulation, impaired RV relaxation and increased mortality in PAH [49]. Consequently, PAH patients with RV failure have an impaired chronotropic response during exercise [55] and an impaired inotropic response to β-adrenergic receptor agonists that seem to be restricted to the RV [46,47,56]. In addition, the activity of key proteins coupling excitation and contraction is reduced in maladaptive RV hypertrophy, which further depresses inotropy. This is typically the case of the ryanodine receptor 2, which controls Ca^++^ effusion from the sarcoplasmic reticulum after cell membrane depolarization and appears polynitrosylated by excessive oxidative stress in failing RVs [27].

### 3.3. RV under Pressure: Heterometric Adaptation and RV Failure

**Figure 3:**
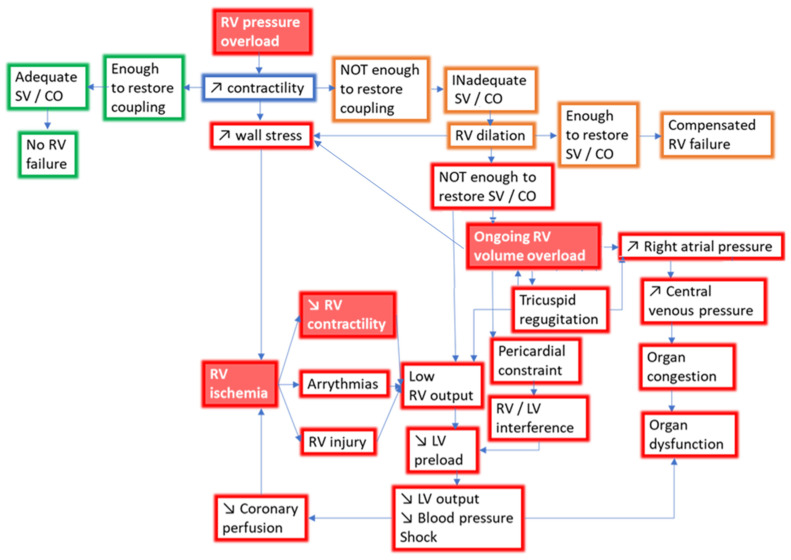
Pathophysiology of RV failure with pressure overload. Red frames indicate adverse events leading to RV failure. Green frames indicate adaptive events improving hemodynamics. Orange frames indicate maladaptive events leading to compensated RV failure. Red labels with white script indicate treatment targets in RV failure. SV, stroke volume; CO, cardiac output; RV, right ventricle; LV, left ventricle.

If RV-PA coupling decreases, the RV may still dilate, attempting to preserve cardiac output through the Frank–Starling mechanism, also known as heterometric adaptation. Of note, the E_es_/E_a_ threshold at which decoupling occurs is unknown, but values between 0.65 and 0.8 have been associated with a worse prognosis in clinical studies [13,57,58,59]. Nevertheless, RV enlargement leads at the same time to profound maladaptive processes that will ultimately result in RV failure and death (Figure 3). In PAH, an increased RV end-diastolic volume, assessed by cardiac magnetic resonance, is indeed a very strong predictor of mortality [53]. 

The pathophysiologic consequences of RV dilatation are numerous. First, the eccentric remodeling of the RV is always accompanied by increased filling pressure due to the stiffening of fibrotic, hypertrophied, maladapted RVs, with a backward increase in RAP and systemic congestion [60]. By definition, this state of RV inability to adequately perfuse the pulmonary circulation (cardiac index < 2.5 L/min/m^2^) without increasing RAP (>8 mmHg) characterizes RV failure [61]. In the setting of left ventricular failure, RAP is the hemodynamic index with the best correlation with end-organ dysfunction, like worsening renal function [62]. In PAH, both decreased cardiac index and increased RAP are independent determinants of worsening renal function over time [63]. Renal congestion resulting from the backward transmission of elevated RAP decreases renal perfusion pressure and the glomerular filtration rate, but it may also compress the tubules, further worsening kidney function [64]. Although most renal failure occurring during acute congestion is functional and reversible, chronic congestion activates the venous endothelium and stimulates local inflammation, in turn triggering definitive glomerular and interstitial structural damage [65].

Functional tricuspid regurgitation (TR) may also occur as a consequence of both RV and RA remodeling, with tricuspid annular dilation, valve tenting and normal leaflets. Besides worsening congestion and RAP, TR also decreases cardiac output. In left heart disease, TR is actually a major determinant of stroke volume [66]. By creating a window towards a vascular circuit with lower resistance and higher compliance than the pulmonary arterial tree, even small orifices may result in an important regurgitation fraction and hence reduced stroke volume in all PH conditions. In PAH, severe TR is quite rare (prevalence < 20%), but it is associated with poor outcomes [67,68]. 

During heterometric adaptation, RV dilatation may still become significant enough to induce pericardial constraint. Competition for space is then triggered between both ventricles, a phenomenon called right–left ventricular interaction. As both cavities share the interventricular septum in addition to the pericardial space, LV transmural pressure may decrease during filling or even invert if RAP is high enough. This causes a mechanical septal leftward shift, leading to LV underfilling and low cardiac output [64]. Indeed, the LV filling rate is well predicted by the leftward interventricular septal curvature, and in turn, stroke volume is much better correlated with LV end-diastolic volume than with RV end-diastolic volume in PAH [69].

Finally, ischemia can severely worsen during heterometric adaptation by enhancing the already existing mismatch between RV myocardial O_2_ demand and delivery, with a further decrease in RV contractility and cardiac output. Indeed, RV dilatation raises parietal tension and reduces wall thickness at the same time, which adversely affects wall stress and O_2_ demand. Simultaneously, the increased RV filling pressure reduces the gradient between aortic diastolic pressure and RV end-diastolic pressure, which may compromise diastolic flow in the right coronary artery and O_2_ delivery. In addition, the already reduced cardiac output and blood pressure may generally jeopardize coronary perfusion in both ventricles [12].

## 4. Assessment of RV-PA Coupling at Bedside

In ambulatory facilities, emergency departments or intensive care units, RV hemodynamics, size and systolic function are usually similarly evaluated by echocardiography at bedside [15]. PH can be assessed with good sensitivity and specificity by the trans-tricuspid velocity gradient converted to pressure according to the simplified Bernoulli equation and by the diameter and respiratory collapse of the inferior vena cava [70]. Many echocardiographic indices are also helpful to estimate RV and RA enlargement as well as RV/LV interference [71]. Unlike LV, RV systolic function can, however, not be estimated by the assessment of the ejection fraction because of its too complex 3-D morphology, but many surrogates like fractional area change (FAC), tricuspid annular plane systolic excursion (TAPSE), S’ velocity of the tricuspid annulus and longitudinal deformation imaging have been developed to overcome this hurdle [15,71]. Of note, all these indices have some flaws, and none of them can be considered to represent contractility or coupling. In recent years, considerable efforts have thus been made to enable a better assessment of coupling, which is undoubtedly the most meaningful parameter to evaluate the severity of RV failure and assess the impact of therapy. 

In addition to non-invasive imaging, right heart catheterization can be needed to assess important hemodynamic aspects addressing RV function like RAP, cardiac output, stroke volume and venous O_2_ saturation. They are all critical prognostic factors in PAH and CTEPH [72,73], and they can help to guide therapy in complex situations, particularly in patients with cardiogenic shock unresponsive to initial treatment [74]. Furthermore, invasive hemodynamics is essential to confirm PH, which is now defined as an mPAP > 20 mmHg at rest, and to discriminate between an elevated mPAP due to left heart disease (with PA wedge pressure > 15 mmHg), pulmonary vascular disease (pulmonary vascular resistance > 2 WU) or a hyperdynamic state with an increased pulmonary blood flow [11]. 

### 4.1. Pressure–Volume Loops

The generation of pressure–volume loops is considered the gold standard method to assess RV-PA coupling. They are best created by the simultaneous high-resolution recording of both parameters with conductance catheters inserted in the right ventricle through the jugular or femoral vein [60]. ESPVR can either be assessed by the generation of a PV loop family during a balloon inflation in the vena cava or during a Valsalva maneuver, or alternatively by the so-called single-beat method. From the pressure curves obtained during the isovolumetric contraction and relaxation phases, this technique first extrapolates the maximal theoretical pressure the RV would produce during a non-ejecting beat with the pulmonary valve hypothetically closed. Then, ESPVR is derived from two pressure–volume points: the E_es_ of the considered single beat and the maximal theoretical E_es_ [17], with good agreement as compared with the gold standard PV loops family model [58]. E_a_ is then calculated by the ratio between end-systolic pressure and stroke volume, and coupling is finally obtained by the ESPVR/E_a_ ratio. 

Both methods were found useful to determine clinical worsening in populations with PAH and CTEPH with an E_es_/E_a_ cut-off between 0.65 and 0.7 [59,75], but as they remain invasive, expensive and inconvenient at bedside, non-invasive surrogates of E_es_/E_a_ have been recently developed. 

### 4.2. Cardiac Magnetic Resonance Imaging

Cardiac magnetic resonance imaging (MRI) is considered the gold standard non-invasive imaging modality for RV volume evaluation [53]. It can also be used to evaluate flow in the PAs, tricuspid regurgitation and tissue characterization by using different acquisition times and techniques (T1, T2, late gadolinium enhancement), but pressure cannot be directly measured. Nevertheless, the E_es_/E_a_ ratio can be simplified for pressure and expressed as a stroke volume/end-systolic volume ratio if the theoretical volume of the unloaded RV is assumed to be null [76], which is actually not the case [77]. In spite of these limitations, volumetric coupling was shown to correlate quite well with E_es_/E_a_ assessed by the pressure–volume curve family model [58] and to predict outcomes better than right ventricular ejection fraction in patients referred for PH investigations (cut-off value at 0.5). Initial RV stroke volume/end-systolic volume <0.53 and decreasing stroke volume/end-systolic volume over time were independently associated with transplant-free survival, but initial RV ejection fraction <0.32 also predicted outcomes [78].

Cardiac MRI remains, however, costly, not broadly available and quite cumbersome in regard to the long time needed to acquire all sequences (about 45 min). Echocardiographic surrogates of E_es_/E_a_ have thus also been developed.

### 4.3. Echocardiography

The ratio of the tricuspid annular plane systolic excursion (TAPSE) to the estimated systolic PAP (sPAP) is certainly the more investigated surrogate of E_es_/E_a_ [60,79]. Described as an index of long axis shortening versus generated force [80], the assumption that TAPSE holds for contractility and sPAP for afterload is incorrect. Nevertheless, the TAPSE/sPAP index has been found to correlate well enough with E_es_/E_a_ (r = 0.498; *p* = 0.001) to be implemented in clinical practice [58]. 

The clinical relevance of the TAPSE/sPAP index was first investigated in patients with left heart disease. In the setting of heart failure with preserved ejection fraction, lower TAPSE/sPAP ratios were associated with worse hemodynamics, worse aerobic maximal capacity and higher NT-proBNP levels [81]. Furthermore, TAPSE/sPAP emerged as the strongest independent predictor of major cardiac events after adjustment for meaningful variables like peak VO_2_, VE/VCO_2_ slope or LV ejection fraction in a mixed population of patients with heart failure and both preserved and reduced ejection fraction [82]. Later, the TAPSE/sPAP ratio was also described as a significant prognostic factor in patients with PAH [83]. Although it displayed a moderate correlation with E_es_/E_a_ in these patients (r = 0.44; *p* = 0.002), a cut-off value <0.31 mm/mmHg identified E_es_/E_a_ <0.805 with quite good predictive values (sensitivity 87.5%, specificity 75.9) and independently predicted a worse outcome [84]. Interestingly, the TAPSE/sPAP ratio combined with the 6 min walking distance also appeared to predict survival with improved accuracy in PAH patients initially classified as intermediate risk by using the NYHA class, 6 min walking distance and NT-proBNP level. Finally, the TAPSE/sPAP ratio was also investigated in the setting of intensive care medicine, where it outperformed conventionally used left-sided cardiac echocardiographic indices to predict successful veno-arterial extracorporeal membrane oxygenation weaning [85].

Of note, numerous other indices like RV fractional area change/mPAP, RV fractional area change/RV end-systolic area and RV longitudinal strain/sPAP were also suggested to represent E_es_/E_a_, but they have been so far less validated than TAPSE/sPAP [15,60,79]. 

## 5. Medical Management of Pressure-Overloaded RV Failure

The management of pressure-overloaded RV failure relies on preload optimization, afterload reduction, maintenance of coronary perfusion and inotrope therapy (Table 1). Acute mechanical circulatory support may be further considered if medical management fails, but only as a bridge to RV failure recovery or lung transplantation. 

### 5.1. Preload and Volume Management

There has been a quite recent change in paradigm regarding preload management in RV failure, emphasizing the importance of not overfilling the patient [12,86]. In acute RV failure, early clinical assessment of volume status is central to adequate management, and invasive monitoring of the RAP should be undertaken if volume status is unclear or if the patient fails to respond to therapy [87]. As RV failure is mostly associated with congestion, diuretics are often considered in the initial steps of management, but it may be acceptable to consider a small intravenous fluid bolus in cases of hypotension. As discussed above, excess preload and filling pressures have the potential to deteriorate end-organ function, worsen tricuspid regurgitation, deteriorate RV-LV interference, worsen ischemia and, finally, reduce cardiac output. They must therefore be aggressively countered. In cases of bi-ventricular failure, diuretics should also aim at reducing left atrial pressure in order to decrease RV pulsatile load [88,89]. 

In patients monitored invasively, there are no specific guidelines on the RAP to target, but values > 8–12 mmHg likely suggest a benefit from decongestion [90]. Fluid removal with intravenous furosemide is initially performed with boluses and continuous infusions at increasing dosage until natriuresis exceeds 50–70 mmol/L and diuresis reaches 100–150 mL/h by analogy with acute left heart failure management [91]. In subjects developing resistance to high-dose loop diuretics, adding a thiazide may augment natriuresis, and the association of carbonic anhydrase inhibitors with loop diuretics was recently shown to improve decongestion in decompensated left heart failure [92]. In volume-overloaded patients with hypotension, diuretics should not be withheld but associated with vasopressors to improve decongestion [86]. Finally, extracorporeal ultrafiltration may be an option to treat patients still resistant to multiple diuretic therapies, but evidence of its benefit on outcome remains as scarce in RV as in LV failure [93]. 

Preload and volume management are also important in chronic progressive RV failure occurring in ambulatory patients, and treatment mostly relies on oral diuretics that must be adjusted according to congestion clinical signs and renal function. As compared to furosemide, torasemide has a more predictable bioavailability and is usually the preferred loop diuretic. Combination therapy with thiazides is sometimes also necessary if resistance to loop diuretics develops [86]. 

### 5.2. Afterload Reduction

The management of RV afterload depends on the underlying cause of RV failure, and evidence-based guidelines are available for many scenarios, such as pulmonary embolism, PAH, CTEPH and left heart disease. On the other hand, management of RV afterload in the setting of critically ill patients in intensive care units may reflect expert opinions more closely.

Patients with acute pulmonary embolism and hemodynamic instability (high-risk patients) should undergo immediate reperfusion therapy, in most cases via systemic thrombolysis, whereas still normotensive patients with signs of RV dysfunction on echocardiography or CT scan (intermediate–high risk) should be monitored for eventual worsening and anticoagulated [94]. 

Occasionally, patients with PAH and CTEPH may be diagnosed with acute RV failure. Parenteral prostanoids should be promptly started and up-titrated in PAH in addition to oral endothelin receptor antagonists, phosphodiesterase type 5 inhibitors or soluble guanylate cyclase stimulators [11]. Of note, previously stable PAH patients with acute RV failure often present treatable precipitating factors like sepsis, supraventricular arrythmias or anemia that should be rapidly diagnosed and treated. In CTEPH, surgical removal of the fibrotic clot residues that characterize the disease should be offered to all patients deemed operable, irrespective of the severity of right heart failure [95]. Indeed, even if NYHA class IV and signs of right heart failure are associated with increased risk and less favorable peri-operative and long-term outcomes [96], the prognosis of operable patients is certainly worse without surgery. On the other hand, patients judged inoperable should be offered balloon pulmonary angioplasty or, alternatively, medical therapy [11].

PH associated with chronic lung disease and/or hypoxia is usually mild to moderate and is rarely at the origin of RV failure [45], except during acute respiratory failure. The therapeutic approaches aimed at reducing afterload include supplementary oxygen and non-invasive ventilation. On the other hand, acute lung injury followed by acute respiratory distress syndrome (ARDS) may be complicated by 25–50% acute RV failure [97,98], and elevated PAP is of poor prognosis in these patients [99]. In ARDS, RV-PA uncoupling may be consecutive to disease-related factors but also to the afterload increase induced by mechanical ventilation [100]. Among the factors related to ARDS severity, sepsis can impair myocardial contractility [101], and hypoxic pulmonary vasoconstriction typically increases RV afterload. Hypoxic PA vasoconstriction occurs in response to decreased partial alveolar O_2_ pressure, but with more intensity if hypercapnia, acidemia and low mixed venous saturation are also present [102,103].

Interventions aiming at relieving RV afterload should ideally correct blood gas and pH imbalance, but these goals may be at odds with the current ventilatory approach in ARDS patients, who benefit at the same time from low-volume ventilation and permissive hypercapnia [104]. In addition to adapted ventilation strategies, inhaled nitric oxide (NO) is also widely used to decrease RV afterload in critically ill patients with RV failure because of its prompt onset of action, short half-time and beneficial effects on arterial and mixed venous O_2_ saturation as well as on RV ejection fraction [105,106]. Nevertheless, NO has not been proven to improve outcomes in ARDS [107].

Finally, in patients presenting with RV failure in addition to LV failure, guidelines regarding acute heart failure therapy should be applied [108]. Drugs approved for PAH are generally neither recommended in PH associated with left heart disease nor in PH associated with chronic lung diseases and may be harmful [11].

### 5.3. Vasopressor Therapy

Fighting RV ischemia is a mainstay of RV failure therapy. Vasopressors are often considered to restore blood pressure and improve blood flow in the right coronary artery. To that end, the ideal vasoactive agent should enhance systemic arterial pressure and at the same time decrease pulmonary vascular resistance, or at least keep it unchanged.

Norepinephrine binds to α_1_ receptors with higher affinity than to β_1_ and exerts a vasoconstricting effect at low doses with weak cardiac inotropy [109]. It is often used as a first-line drug in hypotensive patients with RV failure, but with low grade of evidence [12,86]. In dogs with acute increases in RV afterload, systemic hemodynamics, RV-LV interactions and coronary perfusion gradient can be improved by low-dose norepinephrine without changing pulmonary vascular resistance [110]. However, PA also contains α_1_ receptors, and in patients with sepsis and RV failure, norepinephrine was associated with increased pulmonary vascular resistance and no change in RV ejection fraction despite improvement in RV myocardial O_2_ delivery [111]. 

Low-dose arginine vasopressin (0.01–0.03 U/min) may appear as the most appealing vasopressor in RV failure as it exerts simultaneous systemic vasoconstriction and pulmonary vasodilation (via NO release by endothelial cells) through V1 receptor stimulation [112], thereby decreasing the pulmonary vascular resistance/systemic vascular resistance ratio [113]. At a higher dose (>0.4 U/min), arginine-vasopressin is, however, detrimental in RV failure because it may induce coronary artery vasoconstriction and decrease coronary flow, RV contractility and cardiac output [114]. Currently, the use of arginine-vasopressin is more and more considered for the treatment of persistent pulmonary hypertension in newborns [115], but evidence of its benefit is still scarce in adults with RV failure. 

Importantly, phenylephrine and dopamine are potent constrictors in both pulmonary and systemic circulations, and they are not considered for the treatment of acute RV failure [12,86].

### 5.4. Inotrope Therapy

Inotropic agents can be used in the management of unstable patients with RV failure when there is evidence of persistent inadequate O_2_ delivery despite the optimalization of RV preload and afterload and after the initiation of vasopressors [102]. Of note, a great disparity was observed with regard to the preferred inotropic drug used in RV failure associated with PAH in a recent survey. Whereas 50.4% of PAH centers favor first-line dobutamine, 49.6% prefer milrinone, dopamine or other compounds, highlighting the lack of evidence in the field [116]. 

In anesthetized dogs with acute PA constriction, dobutamine (5–10 μg/kg/min), an agonist of β_1_ and β_2_ receptors, decreased PA resistance and increased RV contractility and RV-PA coupling [117]. However, given the important β_1_ receptor downregulation occurring in chronic RV pressure overload, these observations could not be repeated in chronic RV failure. In experiments performed on rat models with persistent PH induced by various methods, dobutamine-recruitable RV contractile reserve and cAMP concentrations in RV cardiomyocytes were actually severely reduced as compared to controls [118]. On the other hand, clinical data on the hemodynamic effect of β stimulation in RV failure are scarce. In a series of patients with mild-to-moderate PH and end-stage lung disease awaiting lung transplant, Vizza et al. found that dobutamine decreased PVR (through PA β_2_ receptor stimulation [109]) and increased cardiac output [119]. Similar results were also observed in patients with mild PH undergoing orthotopic liver transplantation [120], but their transposition to severe RV failure and severe PH cannot be assumed. Given the high mortality (46%) observed in PAH patients with RV failure treated with catecholamines, β-stimulation may be of little relevance in critical situations [56]. In addition to being relatively inefficient, β_1_ and β_2_ stimulation may still be harmful as they worsen myocardial O_2_ demand and ischemia and trigger arrythmias and hypotension. 

Phosphodiesterase-3 (PDE3) inhibitors like milrinone may appear as a more attractive alternative. Milrinone works by inhibiting the intracellular breakdown of cyclic adenosine monophosphate (cAMP), which promotes Ca^++^ release from the sarcoplasmic reticulum and finally enhances inotropy without β-receptor stimulation [121,122,123]. In PA smooth muscle cells, cAMP is a potent relaxant [124] that is mainly converted from adenosine triphosphate through the stimulation of the prostacyclin pathway and broken down by phosphodiesterase type 3 [125]. By combining RV inotropy with pulmonary vasodilatation, milrinone is thus particularly favorable for restoring RV-PA coupling [126,127]. However, PDE3 inhibitors should be carefully considered and individualized, as they can also cause hypotension and increase the need for concurrent vasopressor support [12]. Although less likely than dobutamine to cause tachycardia [102], PDE3 inhibitors still trigger arrhythmias. Of note, the superiority of milrinone over dobutamine could not be established in the setting of cardiogenic shock occurring from LV failure [128]. 

Belonging to the family of “myofilament Ca^++^ sensitizers”, levosimendan may also be an interesting alternative to dobutamine. Unlike β-adrenoreceptor agonists and PDE3 inhibitors, myofilament Ca^++^ sensitizers do not increase cAMP and Ca^++^ cytosolic concentrations, but they enhance cardiac Troponin C (TnC) sensitivity to Ca^++^ [129]. During systole, levosimendan selectively binds to Ca^++^-associated cardiac TnC and stabilizes the cation–protein link, which triggers more cross-bridging between actin and myosin and generates more force. With this drug, cardiomyocytes need less O_2_ to increase their contractility than with β-adrenoreceptor agonists and PDE3 inhibitors because there is no cytosolic Ca^++^ excess to be actively pumped back into the sarcoplasmic reticulum during diastole, which is a real advantage. 

Besides targeting the heart, levosimendan opens ATP-sensitive K^+^ channels in arterial and venous smooth muscle cells, inducing membrane hyperpolarization and cell relaxation, including in the lung vessels. In various animal models of acute and chronic RV failure and PH, levosimendan was thus consistently shown to restore RV-PA coupling by simultaneously improving RV contractility and reducing RV afterload [130,131,132,133,134,135]. Given that part of levosimendan is converted to a long-lasting active metabolite (OR-1896), these benefits may last for several days after infusion interruption [136]. 

Although clinical data regarding levosimendan accumulate in the fields of septic shock, post-cardiopulmonary bypass low output syndrome and advanced heart failure, there is currently no high-quality randomized controlled trial available for PH-associated RHF. However, numerous small studies reported promising results. In the context of PH associated with left heart disease, levosimendan was consistently associated with reductions in PAP and pulmonary vascular resistance and with the improvement in echocardiographic indices of RV function [137,138,139]. In PAH with severe RV failure, a single infusion of levosimendan improved 6 min walking distance and reduced NTproBNP levels [140]. In a cohort of 87 patients with PAH associated with connective tissue disease, levosimendan (0.1 μg/kg/min during 24 h) resulted in an improvement in mixed venous oxygen saturation, higher TAPSE and lower levels of NT-proBNP compared to enoximone given at 0.5 μg/kg/min [140]. 

Regarding safety, levosimendan was mostly studied in the setting of acute and chronic advanced heart failure. In the landmark SURVIVE and REVIVE trials, systolic and diastolic blood pressure declined significantly more with levosimendan than with comparators (dobutamine or a placebo), which was partly due to a no longer recommended high-dose bolus preceding the 24 h infusions [141,142]. Hypotension was milder when boluses and large doses of loop diuretics were avoided and attention was paid to correcting volume depletion before starting the drug [143]. Nevertheless, systemic vasodilation and hypotension appear to be the major drawbacks of levosimendan use in patients with RV failure. With respect to arrythmias, atrial fibrillation was more frequent with levosimendan than with dobutamine or a placebo in SURVIVE and REVIVE, and a higher rate of ventricular tachycardia was also observed with levosimendan in REVIVE. Of note, repeated levosimendan infusion in chronic advanced heart failure was not associated with more adverse events than a placebo [144,145].

### 5.5. Is There a Place for Mechanical Support in RV Failure?

Mechanical circulatory support (MCS) can be considered if cardiac output remains insufficient to match body O_2_ demand on medical therapy and only as a bridge to recovery or transplantation. Indeed, there is currently no validated option for long-term MCS and destination therapy in RV failure [86,146,147]. 

In cases of significant pulmonary vascular disease, veno-arterial (VA) extracorporeal membrane oxygenation (ECMO) is usually the only possible MCS option [86,146,147]. VA ECMO can be implanted percutaneously or surgically from a peripheral vascular access (groin, neck, sub-clavicular space) at bedside, or alternatively from open chest surgery. Basically, a venous canula is positioned in the right atrium to drain all O_2_-desaturated blood to an oxygenator, and an arterial canula is inserted in the ascending or descending aorta to restitute oxygenated blood to all organs. The benefits of ECMO are numerous: quick installation in emergent situations, rapid correction of hypoxemia and capacity to address both isolated RV failure and bi-ventricular failure if necessary. Whereas devices that increase trans-pulmonary flow (Impella RP, Levitronix...) can be considered in pure RV pump failure (after RV infarction, after left ventricular assist device implantation, after heart transplantation...), they are not contemplated in cases of pulmonary vascular disease because PA pressure may excessively increase and induce vascular lesions and lung hemorrhage [86,146,147]. 

Typical VA-ECMO complications include thrombus formation around canulae, arterial embolism, limb hypoperfusion and local infections that occur increasingly with support duration. In a recent multicentric retrospective evaluation of the outcome of patients treated with VA-ECMO in Germany (43.4% implanted in the context of cardiopulmonary resuscitation), the overall complication rate was 18.4% for bleeding, 0.9% for stroke, 6.6% for abdominal ischemia and 7.6% for limb ischemia [148].

## 6. Perspectives and Conclusions

In recent years, clinicians and scientists have regained interest in RV pathophysiology, and a great deal of effort has been placed on understanding its function in health and disease. Invasive methods were developed to accurately assess its function and adaptation, but the non-invasive evaluation of its coupling to the pulmonary circulation at bedside remains challenging. In addition, therapeutic options aimed at the preservation or improvement in its function principally target pulmonary vessels and not the myocardium. Nevertheless, promising results were also obtained with modulators of metabolism and reactive oxygen species production, as well as with microRNA and long non-coding RNA in animal models of RV failure [149]. In addition, factors identified in the transition from adaptive to maladaptive hypertrophy like altered energy substate metabolism, capillary rarefaction or fibrosis are becoming better understood, as is the tight regulation of gene expression that drives these events. This regulation is typically controlled by multiple types of non-coding RNAs, which may also serve as therapeutic agents or targets to treat RV failure in the future [149,150].

## Figures and Tables

**Table 1:** Hemodynamic effect of right ventricular failure therapies. ↑ indicates an increase, and ↓ indicates a decrease. PVR: pulmonary vascular resistance. SVR: systemic vascular resistance. RV-PA: right ventricle–pulmonary artery. PDE3: phosphodiesterase 3. LV: left ventricle. RAP: right atrial pressure.

	Cardiac Index	PVR	SVR	RV/PA Coupling	RV Ischemia	Remarks
**Volume Depletion**	(↑)	-	-	(↑)	(↓)	Improves end-organ function, RV/LV interference and tricuspid regurgitation Target RAP 8–12 mmHg
**Vasopressors**						
Norepinephrine	↑	↑	↑↑	↑	↓	Often used as first-line therapy
Low-Dose Vasopressin	↑	↓	↑↑	↑	↓	PA vasodilator at low dose
**Inotropes**						
Dobutamine	(↑)	↓	↓	↑	↑	Triggers arrythmias and favors hypotension
PDE3 Inhibitors	↑	↓	↓	↑	↑	Hypotension
Levosimendan	↑	↓	↓	↑	↓	Hypotension

## Data Availability

Not applicable.
